# Conditional survival in patients with esophageal or gastroesophageal junction cancer after receiving various treatment modalities

**DOI:** 10.1002/cam4.3651

**Published:** 2020-12-12

**Authors:** Wei Deng, Zhao Yang, Xin Dong, Rong Yu, Weihu Wang

**Affiliations:** ^1^ Key Laboratory of Carcinogenesis and Translational Research (Ministry of Education/Beijing) Department of Radiation Oncology Peking University Cancer Hospital & Institute Beijing China; ^2^ School of Public Health Li Ka Shing Faculty of Medicine The University of Hong Kong Hong Kong SAR China

**Keywords:** adjusted conditional survival, esophageal cancer, multimodality therapy, SEER database, time‐dependent effect

## Abstract

**Background:**

To estimate the adjusted conditional overall survival (COS) in patients with esophageal cancer after receiving various treatment modalities via a national population‐based database, and to investigate the possible time‐dependent effects.

**Materials and Methods:**

Eligible patients diagnosed with esophageal cancer between 2000 and 2016 were identified from the Surveillance, Epidemiology, and End Results (SEER) registry. The Kaplan‐Meier method was used to calculate conventional survival time. The inverse probability of treatment weighting method was used to estimate the adjusted COS in patients receiving different treatment modalities. Landmark analysis was employed to investigate the possible time‐dependent effects of different treatment modalities in patients who had survived a certain period of time.

**Results:**

A total of 25,232 patients were included in the final analysis. The conventional 5‐year overall survival was 19.3%. The 5‐year adjusted COS increased most for the first 3 years, and increased slightly afterwards. In patients with regional esophageal or gastroesophageal junction cancer, stage‐specific analysis showed that surgery only and preoperative radiation therapy benefited most for patients with localized disease, preoperative radiation therapy plus surgery benefited regional, and preoperative radiation therapy plus surgery benefited distant disease, with the 5‐year adjusted COS given patients had survived 3 years being 67.0% (95% CI 65.2%–68.7%), 59.9% (95% CI 58.3%–61.5%), 58.4% (95% CI 56.3%–60.5%), and 61.8% (95% CI 59.5%–64.1%), respectively. In time‐dependent analysis, the benefits of surgery only in localized cases were prominent within 48 months after diagnosis. Preoperative radiation therapy showed long‐lasting benefits in patients with regional disease. In patients with distant disease, all treatment modalities showed similar and short‐term effects.

**Conclusions:**

The adjusted COS in patients with esophageal cancer increased as time accrued after receiving various treatment modalities. The time‐dependent effects in specific tumor stage provided a dynamic view on optimization of treatment strategies.

## Graphical abstract

The adjusted COS in patients with esophageal cancer increased as time accrued after receiving various treatment modalities. The time‐dependent effects in specific tumor stage provided dynamic view on optimization of treatment strategies.

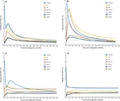

## 1 INTRODUCTION

The 5‐year overall survival of esophageal cancer is poor, which varies from 9% to 22%, and suggests that esophageal cancer, in general, is a fatal neoplasm.^1^, ^2^, ^3^, ^4^, ^5^ In 2018, more than 572,000 new cases were diagnosed with esophageal cancer globally, and about 509,000 cases die from esophageal cancer, accounting for more than 1 in 20 cancer deaths.^6^ Traditionally, only patients with early stage esophageal cancer could receive curative treatment, whereas metastatic, distant nodal disease, or unresectable T4b disease prevents them from undergoing surgical resection or intensive chemoradiation.^7^ Recent advances in treatment modalities, such as the introduction of advanced surgical techniques, adjuvant or neoadjuvant chemoradiation therapy, targeted therapy, and immunotherapy, have been made to improve clinical outcomes of esophageal or gastroesophageal junction cancers. For example, the CROSS trial showed that 49% of patients with esophageal squamous cell carcinoma obtained a pathological complete response after receiving neoadjuvant chemoradiation therapy.^8^ The MAGIC^9^ and the FLOT^10^ trial reported the benefit of perioperative chemotherapy in patients with gastroesophageal junction cancers. Furthermore, the anti‐VEGF receptor antibody ramucirumab in combination with paclitaxel for the second‐line treatment appeared to prolong the overall survival in patients with advanced gastroesophageal junction cancer.^11^ Other recent studies revealed that the programmed cell death‐1 (PD‐1) antibody could benefit patients with advanced esophageal or gastroesophageal junction cancer compared with chemotherapy, especially for those with high PD‐L1 expression.^12^, ^13^, ^14^, ^15^

Existing evidence suggests that the prognosis of patients with esophageal cancer heavily depends on the clinicopathological characteristics and the treatment modalities.^16^ The choices of treatment strategies are based on the accuracy of their estimated prognosis (e.g., median survival time and 5‐year overall survival). However, the conventional survival derived from the Kaplan‐Meier estimator is well‐acknowledged to be less relevant to time elapses after diagnosis, which may fail to reflect the increment in patients' prognosis.^17^ Previous studies suggest that the adjusted conditional overall survival (COS), which refers to the survival probabilities given patients who have survived after a period of time, provides more accurate information about patients' prognosis, especially when the patient exceeds a prespecific landmark time of survival.^18^ Furthermore, the effect of different treatment modalities may change during the follow‐up period. That is, the proportional‐hazards assumption (PHA) may be violated, of which the Cox model may yield the biased effects.

In this study, we assessed the adjusted COS and the time‐dependent effects of various treatment modalities, including surgery, radiation therapy, and chemotherapy, for prognostication in patients with esophageal or gastroesophageal junction cancer using the population‐based SEER (Surveillance, Epidemiology, and End Results) Cancer Statistics Review (CSR) 2000‐2016 dataset.

## 2 METHODS

### 2.1 Design and setting

We performed a population‐based study using data from SEER CSR 2000‐2016 of the National Cancer Institute (released in April 2019). The SEER CSR 2000‐2016 reports the most recent cancer incidence, mortality, survival, prevalence, and lifetime risk statistics from population‐based registries, which covers almost 26% of the US population from 14 regions (greater California, greater Georgia, Metropolitan Atlanta, Metropolitan Detroit, Connecticut, Hawaii, Iowa, New Mexico, Settle [Puget sound], Utah, Alaska, Kentucky, Louisiana, and New Jersey). We extracted data on patient demographics, clinical observations, and prescriptions, which are publicly available on the SEER program.

### 2.2 Study population

Eligible patients were 18 years of age or older with the diagnosis of either squamous cell carcinoma (ICD‐O‐3 codes 8050‐8082) or adenocarcinoma (ICD‐O‐3 codes 8140‐8573) of the esophagus (ICD‐O‐3 for topography codes: C150‐C155, C158‐C159) or the gastroesophageal junction (ICD‐O‐3 for topography codes: C160) between January 2000 and December 2016. Patients were required to have the confirmed diagnosis of esophageal cancer in the primary record via either histology or cytology. Patients who had received any one of chemotherapy, radiation therapy, surgery, or other treatment plans (including patients without any treatment) were permitted to enroll. As such, eight different treatment modalities were constructed, including (1) surgery only, (2) radiation therapy only, (3) chemotherapy only, (4) chemoradiotherapy, (5) preoperative radiation therapy plus surgery, (6) surgery plus postoperative radiation therapy, (7) surgery plus chemotherapy, and (8) no treatment. Patients without the confirmed information about either primary site code or histological code, or with misspecified or unstaged code of localized/regional/distant, or missing values of overall survival information were excluded from the final analysis.

We retrieved demographic characteristics, including age at diagnosis (18–49, 50–59, 60–69, 70–79, 80+ years), sex (male and female), ethnicity (white, black, and others), marital status (single, married, separated/divorced/widowed, and unknown/others), tumor location (esophagus [C150‐C155, C158‐C159] and gastroesophageal junction [C160]), tumor grade (well, moderately, and poorly differentiated, undifferentiated, and cell type undefined) and tumor stage (localized, regional, and distant), and the treatment modalities (surgery only, radiation therapy only, chemotherapy only, chemoradiotherapy, preoperative radiation therapy plus surgery, surgery plus postoperative radiation therapy, surgery plus chemotherapy, and no treatment). The localized tumor stage was defined as tumor confined to the organ of origin; the regional stage was defined as the tumor extended into surrounding organs or tissues, regional lymph nodes, or both; and the distant stage was defined as the tumor spread to distant organs, tissues, or distant lymph nodes. Furthermore, causes of death in patients with esophageal cancer were defined as the presence of the SEER site recode to both cancer and non‐cancer deaths at any time before December 2016.

### 2.3 Case definition

We defined esophageal cancer as a patient with a diagnosis confirmation (based on histology or cytology) of either squamous cell carcinoma or adenocarcinoma of the esophagus or gastroesophageal junction in the primary record in SEER CSR 2000‐2016 dataset.

### 2.4 Outcomes

The primary outcome included all causes of death. COS and hazard ratios (HRs) according to tumor stage (localized, regional, and distant) with an additional adjustment for potential prognostic factors were also reported.

### 2.5 Statistical analysis

The SEER CSR 2000‐2016 dataset coded causes of death; therefore, the time to death was measured from the year of diagnosis to the cause‐specific death. The occurrence was censored if the patient was alive at the end of 31 December 2016. The median follow‐up time was calculated using the reverse Kaplan‐Meier method by flipping the meaning of event and censor, that is, death becomes censor while censor becomes the endpoint of interest.^19^

COS represents the probability of a patient will survival an additional number of years (y), given that the patient has already survived a certain period of years (x) after the diagnosis of esophageal cancer.^17^, ^18^ Intuitively, the 3‐year COS for patients who have survived 5 years can be estimated using the 8‐year survival divided by the 5‐year survival derived from the Kaplan‐Meier estimator. Confidence interval (CI) of COS can also be calculated using a variation of the standard Greenwood formula, as described by Davis et al.^20^ Adjusted COS according to treatment modalities with an additional adjustment for other available prognostic factors in SEER CSR 2000‐2016 was estimated using inverse probability of treatment weighting (IPTW) method.^21^, ^22^

HRs and 95% CIs were estimated using the Cox proportional‐hazards model^23^ to assess time‐independent effects of various treatment modalities, with an additional adjustment for all the publicly available covariates, such as age at diagnosis, sex, ethnicity, marital status, tumor location, grade, and stage. Harrell concordance index (C‐index) and 95% CI were reported to evaluate the performance of the full model with an additional assumption of no cured patients, of which 0.5 represents random change, and the larger the C‐index, the better performance of the model.^24^ The smoothed plots of the weighted Schoenfeld residuals were used to verify the PHA.^25^ When the PHA was not satisfied, landmark analysis approach^26^ was employed to estimate the possible effects of various treatment modalities in patients who had survived for a certain time (i.e., the landmark time), of which the patients who either died or censored before the landmark time or whose follow‐up time was less than the landmark time were excluded. Furthermore, landmark analysis with different landmark times was carried out to bivariate the possible effect of immortal time bias due to the external time period between diagnosis and the time of treatment for these patients receiving at least one treatment.^27^

Furthermore, considering that the population‐based observational study is susceptible to unmeasured or uncontrolled confounding, we further explored the magnitude of the unobserved confounding on the observed treatment‐prognosis association using the E‐value.^28^, ^29^ The E‐value is defined as the minimum strength of association, on the hazard risk scale, that how strong the unmeasured confounding would have to be associated with both the treatment plans and the prognosis for the observed association was required to bias the observed results to the null. With an observed risk ratio of RR or HR with a rare outcome, the E‐value is equal to $RR+\sqrt{RR\times \left(RR‐1\right)}$. In general, the higher the E‐value is, the stronger the unmeasured confounding would be needed to explain away the observed results.^28^

All statistical analyses were carried out using R software (version 3.6.1).^30^ Results were considered significant if a two‐sided *p* < 0.05 was obtained. Lastly, because the study used preexisting data with no personal identifiers, this study was exempt from review by the institutional review board.

## 3 RESULTS

### 3.1 Baseline characteristics

**FIGURE 1:**
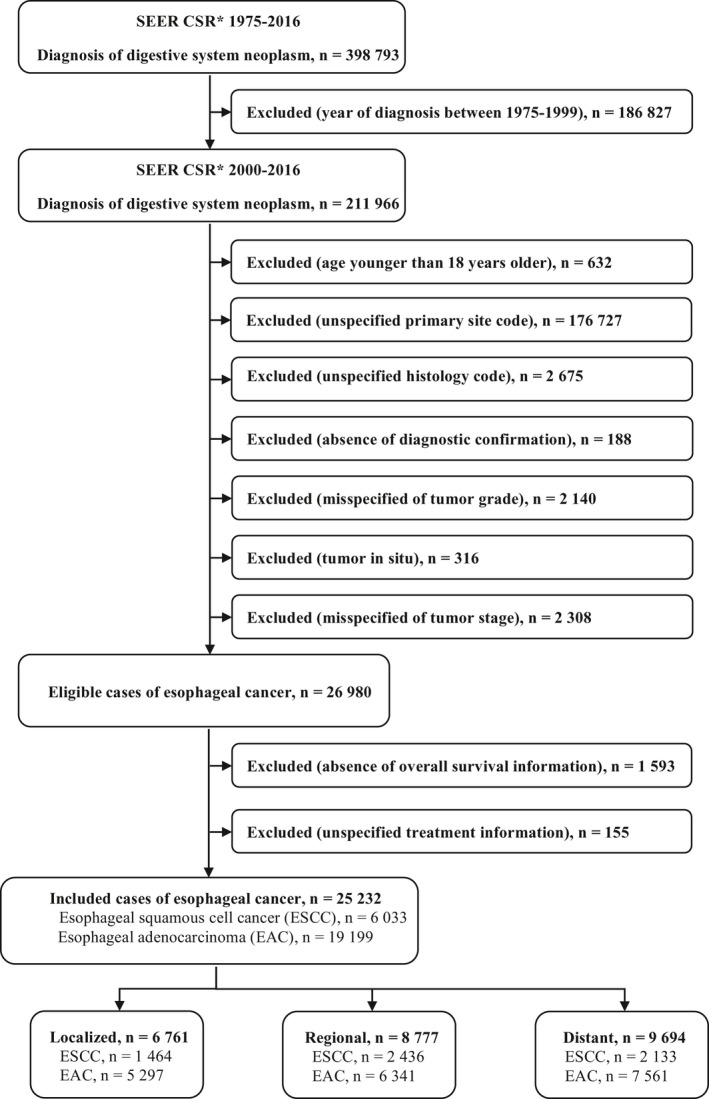
Study flowchart. *Surveillance, Epidemiology, and End Results (SEER) registry program Cancer Statistics Review (CSR)

A total of 25,232 eligible patients, including 19,199 (76.1%) with esophageal adenocarcinoma and 6,033 (23.9%) with esophageal squamous cell carcinoma, were included in the final analysis (Figure 1). Of these, 5,364 (21.3%) patients were female. We categorized the patients into 18–49 years (n = 1,900, 7.5%), 50–59 years (n = 5,070, 20.1%), 60–69 years (n = 7,600, 30.1%), 70–79 years (n = 6,791, 26.9%), and 80 years or older (n = 3,871, 15.3%) based on the age at diagnosis, of which the average age at diagnosis was 66.7 years with standard deviation being 11.9 years.

Table S1 shows the baseline clinicopathological characteristics and causes of death of the eligible patients according to the treatment modalities, in which the bootstrap‐corrected concordance index based on the full Cox proportional‐hazards model was 0.729 (95% CI 0.725–0.733), suggesting that these characteristics could adequately reflect the prognosis of these patients. Among patients receiving at least one treatment, the leading treatment modality was chemoradiotherapy (n = 7,339, 29.1%), followed by preoperative radiation therapy plus surgery (n = 3,624, 14.4%), surgery only (n = 3,453, 13.7%), and chemotherapy only (n = 3,265, 12.9%). Surgery (n = 2,407, 35.6%) was the most prevalent treatment modality for patients with localized disease. For patients with regional disease, chemoradiotherapy (n = 2,792, 31.8%) or preoperative radiation therapy plus surgery (n = 2,366, 27.0%) was the principal treatment modality. In addition, chemoradiotherapy (n = 2,970, 30.6%) or chemotherapy (n = 2,745, 28.3%) was the leading treatment modality for patients with distant diseases. These results were expected under the National Comprehensive Cancer Network guidelines for esophageal and gastroesophageal junction cancers. Among these patients, the leading cause of death was esophageal cancer (n = 13,814, 66.9%), and then followed by stomach cancer (n = 2,771, 13.4%) and heart diseases (n = 861, 4.2%).

### 3.2 Adjusted conditional overall survival (COS)

The median follow‐up time for the eligible patients was 91.0 months (95% CI 88.7–93.3 months). Among the eligible patients, the median survival time was 13.0 months, with the 5‐year overall survival being 19.3% (95% CI 18.8%–19.9%). The 5‐year COS for patients who had already survived 1, 2, 3, 4, and 5 years were 65.7% (95% CI 65.2%–66.3%), 76.8% (95% CI 76.2%–77.4%), 83.2% (95% CI 82.6%–83.7%), 87.1% (95% CI 86.5%–87.6%), and 89.4% (95% CI 88.9%–90.0%), respectively, after adjustment for clinicopathological factors. Considering that the adjusted COS given the patients who have survived 3 years was stable afterwards, we regarded this COS as the main result. Such stable COS also suggested that there might exist a fraction of the patients who were cured.

Overall, patients receiving preoperative radiation therapy plus surgery had the best prognosis with the 5‐year adjusted COS given the patients have survived 3 years being 59.9% (95% CI 58.7%–61.0%), followed by the patients receiving surgery only (57.9%, 95% CI 56.5%–59.2%), and receiving surgery plus postoperative radiation therapy (48.0%, 95% CI 46.7%–49.3%). In addition, patients receiving chemotherapy only had the worst prognosis with the 5‐year adjusted COS given the patients have survived 3 years being 29.9% (95% CI 27.3%–32.6%). For patients without receiving any treatment, the 5‐year adjusted COS given that the patients have already survived 3 years was 62.2% (95% CI 59.7%–64.6%), respectively, which suggested that there might be a small portion of patients who were misclassified to be esophageal cancer (Table S2).

Table 1 presents the stage‐specific conditional survival at various time points stratified by the treatment modality after adjusting for all available covariates. For patients with localized disease receiving at least one treatment, those who had surgery only had the best prognosis, with the 5‐year adjusted COS given patients had survived 3 years being 67.0% (95% CI 65.2%–68.7%) in terms of those of other treatment plans. In patients with regional esophageal or gastroesophageal junction cancer, preoperative radiation therapy plus surgery benefited patients the most with a 5‐year adjusted COS given patients had survived 3 years being 58.4% (95% CI 56.3%–60.5%), and then followed by surgery plus chemotherapy and surgery plus postoperative radiation therapy with the 5‐year adjusted COS given patients had survived 3 years of 51.4% (95% CI 49.1%–53.6%) and 48.6% (95% CI 46.6%–50.7%), respectively. Patients receiving chemotherapy only had the worst prognosis. Finally, in patients with distant disease, the preoperative radiation therapy plus surgery would benefit the most with 5‐year adjusted COS after patients who have already survived 3 years being 61.8% (95% CI 59.5%–64.1%), which were better than any of other treatment plans.

**TABLE 1:** Conditional probabilities of stage‐specific overall survival at various time points stratified by treatment modalities after adjustment for age at diagnosis, sex, ethnicity, marital status, tumor grade and stage, histological type, tumor location, and year at diagnosis

	Time point (months) by treatment modalities^a^	Conditional probability of survival (%) by time point (months)					
		36	48	60	72	84	96
Localized	None						
	12	62.1 (60.2–64.0)	51.5 (49.5–53.5)	48.6 (46.6–50.6)	46.4 (44.3–48.4)	43.2 (41.1–45.3)	41.7 (39.6–43.9)
	24	84.7 (82.9–86.4)	70.2 (68.0–72.4)	66.3 (64.0–68.5)	63.3 (60.8–65.6)	58.9 (56.3–61.3)	56.9 (54.3–59.5)
	36	100 (100–100)	82.9 (80.8–84.8)	78.3 (75.9–80.4)	74.6 (72.2–76.9)	69.5 (66.8–72.0)	67.2 (64.3–69.8)
	S						
	12	83.2 (82.1–84.2)	76.8 (75.6–77.9)	71.1 (69.8–72.3)	66.3 (64.9–67.6)	60.1 (58.5–61.5)	55.7 (54.1–57.3)
	24	91.6 (90.8–92.4)	84.6 (83.5–85.6)	78.3 (77.0–79.6)	73.0 (71.6–74.4)	66.2 (64.6–67.7)	61.4 (59.7–63.0)
	36	100 (100–100)	92.3 (91.4–93.1)	85.5 (84.3–86.6)	79.7 (78.3–81.0)	72.2 (70.6–73.8)	67.0 (65.2–68.7)
	RT						
	12	34.0 (32.1–35.9)	26.9 (25.2–28.7)	19.4 (17.8–21.0)	16.6 (15.1–18.2)	13.3 (11.9–14.8)	13.0 (11.6–14.6)
	24	65.2 (62.4–67.8)	51.7 (48.8–54.4)	37.2 (34.4–40.0)	31.9 (29.2–34.6)	25.6 (22.9–28.3)	25.0 (22.3–27.7)
	36	100 (100–100)	79.2 (76.0–82.1)	57.1 (53.3–60.6)	48.9 (45.1–52.5)	39.2 (35.4–43.0)	38.3 (34.5–42.2)
	CT						
	12	27.9 (26.2–29.5)	24.0 (22.5–25.6)	17.6 (16.2–19.1)	11.5 (10.2–12.8)	11.5 (10.2–12.8)	10.2 (9.0–11.5)
	24	47.2 (44.7–49.6)	40.7 (38.3–43.1)	29.9 (27.6–32.2)	19.4 (17.4–21.5)	19.4 (17.4–21.5)	17.3 (15.3–19.3)
	36	100 (100–100)	86.2 (83.5–88.6)	63.3 (59.5–66.7)	41.1 (37.3–44.9)	41.1 (37.3–44.9)	36.6 (32.7–40.4)
	RT+CT						
	12	46.6 (44.9–48.2)	37.1 (35.4–38.7)	29.3 (27.8–30.9)	22.9 (21.5–24.4)	19.2 (17.8–20.6)	16.7 (15.4–18.1)
	24	72.3 (70.3–74.2)	57.5 (55.4–59.6)	45.5 (43.3–47.7)	35.6 (33.5–37.7)	29.7 (27.7–31.8)	25.9 (23.9–28.0)
	36	100 (100–100)	79.6 (77.4–81.6)	63.0 (60.4–65.4)	49.3 (46.6–51.9)	41.2 (38.5–43.8)	35.9 (33.2–38.5)
	RT+S						
	12	73.7 (72.6–74.8)	63.0 (61.8–64.3)	58.0 (56.7–59.3)	56.0 (54.7–57.3)	47.1 (45.7–48.4)	44.2 (42.8–45.5)
	24	87.6 (86.7–88.5)	74.9 (73.7–76.1)	69.0 (67.6–70.3)	66.6 (65.2–67.9)	55.9 (54.4–57.4)	52.5 (50.9–54.0)
	36	100 (100–100)	85.5 (84.4–86.5)	78.7 (77.4–80.0)	76.0 (74.6–77.3)	63.8 (62.2–65.4)	59.9 (58.3–61.5)
	S+RT						
	12	64.6 (63.3–65.8)	55.9 (54.5–57.2)	44.8 (43.4–46.2)	40.2 (38.8–41.6)	36.0 (34.6–37.4)	32.7 (31.3–34.1)
	24	87.7 (86.5–88.8)	75.9 (74.4–77.3)	60.9 (59.2–62.5)	54.5 (52.8–56.3)	48.9 (47.1–50.6)	44.4 (42.6–46.2)
	36	100 (100–100)	86.5 (85.2–87.7)	69.4 (67.6–71.1)	62.2 (60.3–64.0)	55.7 (53.8–57.6)	50.6 (48.7–52.6)
	S+CT						
	12	77.0 (75.9–78.1)	53.7 (52.4–55.0)	45.5 (44.2–46.8)	45.2 (43.8–46.5)	33.0 (31.7–34.3)	25.7 (24.4–27.0)
	24	88.0 (87.1–88.9)	61.3 (59.9–62.7)	52.0 (50.5–53.4)	51.6 (50.2–53.0)	37.7 (36.2–39.1)	29.4 (27.9–30.8)
	36	100 (100–100)	69.7 (68.3–71.0)	59.1 (57.5–60.6)	58.6 (57.1–60.1)	42.8 (41.2–44.4)	33.4 (31.8–35.0)
Regional	None						
	12	26.3 (24.1–28.6)	19.5 (17.4–21.7)	18.0 (15.9–20.2)	14.4 (12.5–16.4)		
	24	71.9 (67.7–75.6)	53.3 (48.5–57.9)	49.1 (44.3–53.8)	39.3 (34.5–44.0)		
	36	100 (100–100)	74.2 (68.6–78.9)	68.3 (62.5–73.5)	54.6 (48.5–60.3)		
	S						
	12	46.0 (44.6–47.4)	40.2 (38.8–41.6)	33.7 (32.4–35.1)	28.1 (26.8–29.4)	25.0 (23.8–26.3)	22.5 (21.2–23.8)
	24	74.6 (72.9–76.3)	65.2 (63.4–67.0)	54.7 (52.8–56.6)	45.6 (43.7–47.5)	40.6 (38.7–42.5)	36.5 (34.6–38.4)
	36	100 (100–100)	87.4 (85.7–88.8)	73.3 (71.3–75.3)	61.1 (58.8–63.3)	54.4 (52.1–56.7)	48.9 (46.5–51.2)
	RT						
	12	33.5 (31.3–35.6)	18.0 (16.3–19.8)	18.0 (16.3–19.8)	13.2 (11.7–14.8)	13.2 (11.7–14.8)	12.6 (11.1–14.2)
	24	71.7 (68.5–74.7)	38.6 (35.2–41.9)	38.6 (35.2–41.9)	28.3 (25.2–31.4)	28.3 (25.2–31.4)	27.0 (24.0–30.1)
	36	100 (100–100)	53.8 (50.0–57.8)	53.8 (49.7–57.8)	39.5 (35.5–43.4)	39.5 (35.5–43.4)	37.6 (33.7–41.6)
	CT						
	12	26.1 (24.5–27.7)	22.7 (21.1–24.3)	17.9 (16.4–19.5)	17.9 (16.4–19.5)	8.7 (7.4–10.1)	
	24	63.0 (59.9–65.9)	54.8 (51.7–57.9)	43.3 (40.0–46.5)	43.3 (40.0–46.5)	21.0 (17.9–24.2)	
	36	100 (100–100)	87.0 (84.1–89.5)	68.7 (64.5–72.5)	68.7 (64.5–72.5)	33.3 (28.6–38.0)	
	RT+CT						
	12	38.2 (36.7–39.7)	29.8 (28.3–31.3)	23.7 (22.3–25.2)	19.8 (18.4–21.2)	17.1 (15.7–18.5)	15.2 (13.9–16.6)
	24	70.3 (68.1–72.3)	54.8 (52.5–57.1)	43.7 (41.3–46.0)	36.4 (34.1–38.8)	31.4 (29.0–33.8)	27.9 (25.6–30.3)
	36	100 (100–100)	78.0 (75.5–80.3)	62.1 (59.2–64.9)	51.8 (48.8–54.8)	44.7 (41.5–47.7)	39.7 (36.6–42.9)
	RT+S						
	12	56.6 (55.3–57.9)	47.7 (46.4–49.0)	42.5 (41.1–43.8)	37.6 (36.3–39.0)	34.9 (33.5–36.2)	33.1 (31.7–34.5)
	24	79.8 (78.4–81.0)	67.3 (65.7–68.8)	59.9 (58.2–61.5)	53.1 (51.3–54.8)	49.2 (47.4–50.9)	46.6 (44.8–48.4)
	36	100 (100–100)	84.3 (82.9–85.7)	75.0 (73.3–76.7)	66.5 (64.6–68.4)	61.6 (59.6–63.6)	58.4 (56.3–60.5)
	S+RT						
	12	53.0 (51.7–54.4)	43.8 (42.4–45.1)	36.5 (35.2–37.8)	30.6 (29.3–31.9)	27.4 (26.1–28.7)	25.8 (24.5–27.1)
	24	76.8 (75.4–78.2)	63.4 (61.8–65.0)	52.9 (51.2–54.5)	44.3 (42.6–46.0)	39.7 (38.0–41.4)	37.4 (35.6–39.1)
	36	100 (100–100)	82.5 (81.0–83.9)	68.8 (67.0–70.6)	57.7 (55.7–59.6)	51.7 (49.6–53.7)	48.6 (46.6–50.7)
	S+CT						
	12	53.5 (52.1–54.8)	46.5 (45.1–47.8)	39.7 (38.4–41.1)	35.4 (34.1–36.8)	31.0 (29.7–32.4)	27.5 (26.1–28.9)
	24	68.7 (67.3–70.1)	59.7 (58.2–61.2)	51.1 (49.5–52.6)	45.6 (43.9–47.2)	39.9 (38.2–41.5)	35.3 (33.6–37.0)
	36	100 (100–100)	86.9 (85.5–88.1)	74.3 (72.6–76.0)	66.3 (64.3–68.1)	58.0 (55.9–60.1)	51.4 (49.1–53.6)
Distant	None						
	12	23.8 (20.6–27.2)	16.8 (13.9–20.0)	15.1 (12.3–18.2)	12.1 (9.5–15.1)	10.1 (7.6–13.0)	
	24	66.5 (59.7–72.4)	46.8 (39.7–53.6)	42.1 (35.0–48.9)	33.8 (26.9–40.9)	28.1 (21.3–35.3)	
	36	100 (100–100)	70.5 (61.5–77.7)	63.3 (54.0–71.2)	50.9 (41.1–59.9)	42.3 (32.5–51.8)	
	S						
	12	21.3 (19.8–22.8)	12.7 (11.6–14.0)	10.3 (9.2–11.4)	10.3 (9.2–11.4)	7.7 (6.7–8.7)	7.7 (6.7–8.7)
	24	59.3 (56.1–62.3)	35.5 (32.5–38.4)	28.6 (25.9–31.4)	28.6 (25.9–31.4)	21.4 (18.8–24.1)	21.4 (18.8–24.1)
	36	100 (100–100)	59.8 (55.8–63.6)	48.3 (44.2–52.2)	48.3 (44.2–52.2)	36.1 (32.1–40.1)	36.1 (32.1–40.1)
	RT						
	12	27.2 (23.7–30.9)	11.3 (8.9–14.0)	9.5 (7.3–12.0)	9.5 (7.3–12.0)	4.0 (2.6–5.8)	
	24	80.3 (74.1–85.1)	33.4 (26.9–39.9)	27.9 (21.9–34.3)	27.9 (21.9–34.3)	11.7 (7.5–16.8)	
	36	100 (100–100)	41.6 (33.9–49.1)	34.8 (27.5–42.2)	34.8 (27.5–42.2)	14.5 (9.4–20.7)	
	CT						
	12	14.1 (12.8–15.5)	9.6 (8.4–10.8)	6.3 (5.3–7.4)	5.5 (4.5–6.6)	4.3 (3.4–5.4)	4.3 (3.4–5.4)
	24	40.2 (36.8–43.5)	27.2 (24.1–30.4)	17.9 (15.1–20.9)	15.6 (12.9–18.6)	12.4 (9.8–15.2)	12.4 (9.8–15.2)
	36	100 (100–100)	67.8 (61.7–73.2)	44.6 (38.3–50.8)	38.9 (32.5–45.1)	30.8 (24.7–37.1)	30.8 (24.7–37.1)
	RT+CT						
	12	22.5 (21.0–23.9)	15.2 (13.9–16.5)	12.7 (11.5–13.9)	11.0 (9.9–12.2)	9.6 (8.5–10.8)	9.3 (8.3–10.5)
	24	54.3 (51.5–57.1)	36.7 (34.0–39.5)	30.7 (28.1–33.4)	26.7 (24.1–29.4)	23.3 (20.7–25.9)	22.6 (20.1–25.2)
	36	100 (100–100)	67.6 (63.6–71.3)	56.6 (52.4–60.5)	49.2 (44.9–53.3)	42.8 (38.5–47.0)	41.6 (37.3–45.8)
	RT+S						
	12	52.4 (51.1–53.6)	45.3 (44.1–46.6)	40.6 (39.3–42.0)	37.5 (36.1–38.8)	34.1 (32.7–35.5)	32.4 (31.0–33.8)
	24	81.1 (79.8–82.3)	70.2 (68.6–71.6)	62.9 (61.2–64.6)	58.0 (56.2–59.8)	52.8 (50.8–54.7)	50.2 (48.2–52.1)
	36	100 (100–100)	86.5 (85.1–87.8)	77.6 (75.8–79.3)	71.5 (69.4–73.4)	65.0 (62.8–67.2)	61.8 (59.5–64.1)
	S+RT						
	12	33.9 (32.5–35.4)	23.6 (22.3–24.9)	21.2 (19.9–22.5)	16.7 (15.5–17.9)	15.2 (14.0–16.4)	13.5 (12.4–14.8)
	24	66.2 (64.0–68.2)	46.0 (43.8–48.2)	41.3 (39.1–43.5)	32.5 (30.3–34.8)	29.6 (27.4–31.8)	26.4 (24.2–28.6)
	36	100 (100–100)	69.5 (66.9–72.0)	62.5 (59.7–65.1)	49.2 (46.2–52.1)	44.7 (41.7–47.7)	39.9 (36.8–42.9)
	S+CT						
	12	34.2 (32.9–35.6)	26.1 (24.8–27.4)	21.5 (20.3–22.8)	18.2 (17.0–19.4)	17.1 (15.9–18.4)	
	24	60.3 (58.3–62.2)	46.0 (43.9–47.9)	37.9 (35.9–39.9)	32.0 (30.0–34.0)	30.2 (28.2–32.2)	
	36	100 (100–100)	76.2 (73.8–78.5)	62.9 (60.2–65.4)	53.1 (50.1–55.9)	50.1 (47.1–53.0)	

^a^ S, surgery only; RT, radiation therapy only; CT, chemotherapy only; RT+CT, chemoradiotherapy; RT+S, preoperative radiation therapy plus surgery; S+RT, surgery plus postoperative radiation therapy; S+CT, surgery plus chemotherapy; None: no treatment.

### 3.3 Time‐dependent effects of various treatment modalities

**FIGURE 2:**
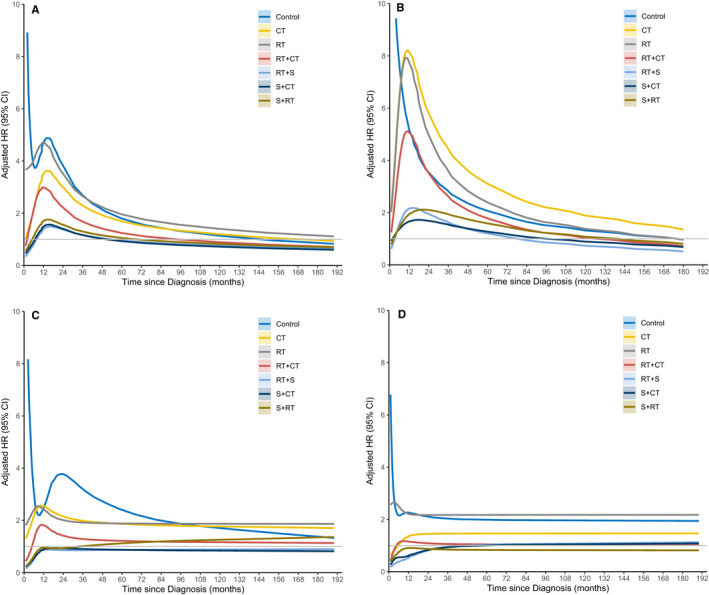
Time‐dependent effects of various treatment modalities with additional adjustment for all available prognostic factors in (A) all patients, (B) patients with localized disease, (C) patients with regional disease, and (D) patients with distant disease

We investigated the possible time‐dependent effects of various treatment modalities in patients with different stages with (Figure 2) additional adjustment for all available prognostic factors. Of these, patients receiving surgery only was considered as the reference group. It is evident that the effects of these treatment modalities were heavily dependent on the clinicopathological characteristics and varied during the follow‐up period. This was also indicated by the Schoenfeld residual tests (*p* < 0.001 for all treatment modalities except for no treatment with *p* = 0.084).

Among all the patients, as a general trend, the HRs increased at first and reached the peak within the first 2 years, and then dropped steadily (Figure 2A). Without considering time‐dependent effect, in comparison with surgery only, patients receiving preoperative radiation therapy plus surgery benefited the most, with an HR of 0.88 (95% CI 0.83–0.94). Moreover, this observed effect is less likely to be subject to the unmeasured confounding with an E value of 1.53. That is, if there existed an unmeasured confounding that would bias away from the observed results to the null, the minimum magnitude of associations of the unmeasured confounding with both the treatment plans and the death would be 1.53. Moreover, the results from the landmark analysis with various landmark time (Table 2) showed that patients receiving surgery only would benefit the most, and this benefit persisted until 48 months after diagnosis, and then was comparable with those receiving either preoperative radiation therapy plus surgery or surgery plus chemotherapy with the HRs being 1.06 (95% CI 0.91–1.24) and 1.04 (95% CI 0.79–1.38), respectively. Such results indicated that the time‐dependent effects of different treatment modalities existed in this study. Furthermore, similar results were observed in patients with localized disease (Figure 2B). Of these, surgery only was the best treatment modality, especially for the first 48 months follow‐up. Afterward, patients receiving preoperative radiation therapy plus surgery and surgery plus chemotherapy would have a comparable prognosis, with the HRs being 1.22 (95% CI 0.98–1.52) and 1.10 (95% CI 0.60–2.00).

**TABLE 2:** Hazard ratio and 95% confidence interval with the E‐value with different landmark time stratified by stage after adjustment for age at diagnosis, sex, ethnicity, marital status, tumor grade and stage, histological type, tumor location, and year at diagnosis

Treatment modalities^a^	Hazard ratio (95% confidence interval)/E‐value with different landmark time (LT)					
	LT = 0	LT = 12	LT = 24	LT = 36	LT = 48	LT = 60
Total						
S	1	1	1	1	1	1
RT	3.65 (3.40–3.91)/6.76	2.91 (2.55–3.31)/5.26	2.59 (2.13–3.16)/4.62	2.53 (1.94–3.29)/4.49	2.07 (1.45–2.96)/3.56	2.10 (1.37–3.22)/3.62
CT	2.14 (2.01–2.29)/3.71	2.94 (2.66–3.25)/5.33	3.17 (2.71–3.70)/5.79	2.17 (1.68–2.79)/3.76	2.23 (1.60–3.11)/3.89	1.51 (0.94–2.43)/2.40
RT+CT	1.81 (1.72–1.91)/3.03	2.11 (1.95–2.27)/3.63	1.92 (1.73–2.12)/3.25	1.75 (1.54–1.99)/2.89	1.62 (1.39–1.89)/2.63	1.45 (1.20–1.74)/2.25
RT+S	0.88 (0.83–0.94)/1.53	1.16 (1.07–1.26)/1.60	1.19 (1.07–1.33)/1.67	1.14 (1.00–1.30)/1.54	1.06 (0.91–1.24)/1.32	1.04 (0.87–1.25)/1.24
S+RT	1.14 (1.06–1.22)/1.53	1.40 (1.28–1.54)/2.16	1.43 (1.27–1.62)/2.22	1.43 (1.23–1.66)/2.21	1.42 (1.18–1.70)/2.19	1.40 (1.13–1.73)/2.14
S+CT	0.97 (0.88–1.08)/1.19	1.25 (1.10–1.43)/1.82	1.27 (1.07–1.51)/1.86	1.16 (0.93–1.45)/1.59	1.04 (0.79–1.38)/1.26	1.04 (0.75–1.46)/1.26
None	4.52 (4.25–4.79)/8.50	2.16 (1.93–2.42)/3.75	1.64 (1.39–1.94)/2.66	1.59 (1.28–1.96)/2.55	1.16 (0.87–1.55)/1.59	1.16 (0.82–1.63)/1.59
Local						
S	1	1	1	1	1	1
RT	4.13 (3.65–4.66)/7.72	4.01 (3.35–4.81)/7.49	3.14 (2.40–4.11)/5.73	2.33 (1.60–3.39)/4.09	2.49 (1.61–3.85)/4.42	2.10 (1.20–3.68)/3.63
CT	4.65 (3.99–5.42)/8.77	4.71 (3.75–5.92)/8.89	4.27 (3.07–5.93)/8.01	2.51 (1.43–4.42)/4.46	3.06 (1.59–5.88)/5.57	2.05 (0.82–5.14)/3.51
RT+CT	2.78 (2.55–3.03)/5.00	2.69 (2.41–3.00)/4.82	2.29 (2.00–2.64)/4.02	1.99 (1.68–2.36)/3.40	1.94 (1.59–2.38)/3.30	1.75 (1.37–2.24)/2.90
RT+S	1.40 (1.25–1.57)/2.15	1.49 (1.31–1.71)/2.35	1.38 (1.18–1.62)/2.11	1.32 (1.10–1.59)/1.97	1.22 (0.98–1.52)/1.74	1.19 (0.92–1.53)/1.66
S+RT	1.52 (1.28–1.81)/2.41	1.75 (1.44–2.13)/2.89	1.58 (1.24–2.01)/2.54	1.58 (1.20–2.08)/2.53	1.60 (1.16–2.19)/2.57	1.53 (1.06–2.21)/2.43
S+CT	1.35 (1.04–1.76)/2.05	1.46 (1.07–1.98)/2.27	1.41 (0.97–2.06)/2.18	1.39 (0.89–2.18)/2.13	1.10 (0.60–2.00)/1.42	1.32 (0.68–2.57)/1.96
None	4.66 (4.25–5.11)/8.79	2.38 (2.05–2.75)/4.18	1.83 (1.50–2.23)/3.06	1.73 (1.36–2.20)/2.86	1.30 (0.94–1.79)/1.92	1.25 (0.86–1.82)/1.81
Regional						
S	1	1	1	1	1	1
RT	2.16 (1.91–2.44)/3.74	1.72 (1.37–2.15)/2.83	1.80 (1.28–2.52)/2.99	2.06 (1.30–3.24)/3.53	1.08 (0.52–2.26)/1.38	1.61 (0.76–3.40)/2.59
CT	2.08 (1.81–2.40)/3.58	1.94 (1.51–2.48)/3.28	1.60 (1.05–2.45)/2.59	1.29 (0.68–2.47)/1.91	1.23 (0.57–2.66)/1.75	1.19 (0.47–2.99)/1.67
RT+CT	1.25 (1.15–1.37)/1.82	1.40 (1.24–1.58)/2.15	1.29 (1.08–1.53)/1.90	1.28 (1.02–1.60)/1.88	1.09 (0.84–1.43)/1.42	0.96 (0.69–1.33)/1.27
RT+S	0.63 (0.58–0.69)/2.54	0.82 (0.72–0.93)/1.74	0.84 (0.71–1.00)/1.66	0.80 (0.65–1.00)/1.80	0.71 (0.55–0.91)/2.17	0.72 (0.53–0.97)/2.12
S+RT	0.75 (0.68–0.84)/1.98	0.94 (0.82–1.08)/1.31	1.02 (0.85–1.22)/1.18	1.06 (0.84–1.33)/1.30	0.98 (0.76–1.28)/1.14	0.97 (0.70–1.33)/1.23
S+CT	0.68 (0.60–0.78)/2.29	0.86 (0.73–1.03)/1.59	0.91 (0.72–1.15)/1.42	0.81 (0.59–1.10)/1.78	0.72 (0.50–1.04)/2.12	0.74 (0.47–1.15)/2.06
None	3.42 (3.06–3.83)/6.30	2.06 (1.62–2.61)/3.54	1.20 (0.75–1.93)/1.69	0.88 (0.43–1.81)/1.54	0.54 (0.20–1.49)/3.09	0.54 (0.17–1.75)/3.08
Distant						
S	1	1	1	1	1	1
RT	2.41 (2.03–2.86)/4.25	1.55 (1.04–2.32)/2.47	1.31 (0.63–2.73)/1.95	1.80 (0.69–4.70)/3.00	1.28 (0.27–6.08)/1.89	1.88 (0.26–13.45)/3.17
CT	1.02 (0.87–1.20)/1.16	1.33 (0.98–1.81)/1.99	1.66 (0.97–2.83)/2.70	1.10 (0.53–2.26)/1.42	0.94 (0.34–2.56)/1.33	0.62 (0.16–2.42)/2.63
RT+CT	0.90 (0.77–1.06)/1.46	1.05 (0.78–1.43)/1.30	1.12 (0.66–1.90)/1.48	0.83 (0.41–1.68)/1.69	0.60 (0.23–1.57)/2.72	0.67 (0.19–2.35)/2.36
RT+S	0.39 (0.32–0.47)/4.56	0.53 (0.38–0.73)/3.18	0.61 (0.35–1.06)/2.65	0.56 (0.27–1.15)/2.98	0.48 (0.18–1.29)/3.57	0.62 (0.18–2.21)/2.59
S+RT	0.68 (0.57–0.82)/2.29	0.81 (0.58–1.13)/1.77	0.89 (0.51–1.55)/1.51	0.74 (0.36–1.55)/2.04	0.60 (0.22–1.64)/2.72	0.90 (0.25–3.25)/1.46
S+CT	0.52 (0.41–0.65)/3.27	0.67 (0.46–0.97)/2.36	0.67 (0.36–1.27)/2.33	0.66 (0.28–1.52)/2.42	0.54 (0.17–1.73)/3.13	0.51 (0.11–2.45)/3.35
None	2.75 (2.34–3.24)/4.95	1.13 (0.78–1.62)/1.51	0.94 (0.48–1.81)/1.34	0.95 (0.38–2.34)/1.30	0.49 (0.12–1.99)/3.51	0.58 (0.10–3.36)/2.81

^a^ S, surgery only; RT, radiation therapy only; CT, chemotherapy only; RT+CT, chemoradiotherapy; RT+S, preoperative radiation therapy plus surgery; S+RT, surgery plus postoperative radiation therapy; S+CT, surgery plus chemotherapy; None, no treatment.

In contrast, for patients with regional disease, preoperative radiation therapy plus surgery benefited the patients most (Figure 2C), and the results derived from landmark analysis showed that this effect lasted for a long time (landmark time = 12 months: HR 0.82, 95% CI 0.72–0.93; landmark time = 24 months: HR 0.84, 95% CI 0.71–1.00; landmark time = 36 months: HR 0.80, 95% CI 0.65–1.00; landmark time = 48 months: HR 0.71, 95% CI 0.55–0.91; landmark time = 60 months: HR 0.72, 95% CI 0.53–0.97). Moreover, surgery plus chemotherapy and surgery plus postoperative radiation therapy showed comparable effects with surgery only. Finally, in patients with distant disease, preoperative radiation therapy plus surgery and surgery plus chemotherapy would benefit the patients best in comparison with surgery only (Figure 2D), especially for the first 12 months after diagnosis with the HRs at landmark time of 12 months being 0.53 (95% CI 0.38–0.73) and 0.67 (95% CI 0.46–0.97), respectively. Afterward, no significant differences in patients receiving these treatment modalities were observed.

## 4 DISCUSSION

Our population‐based analysis showed that patients with esophageal or gastroesophageal junction cancer would have increased COS as time accrued. In detail, for patients with localized disease, surgery only benefited patients the most, especially for the first 48 months after diagnosis. However, for patients with regional disease, preoperative radiation therapy plus surgery appeared to be the optimal treatment strategy. Furthermore, for those patients with distant disease, preoperative radiation therapy plus surgery and surgery plus chemotherapy showed the benefits during the first 12 months after diagnosis in comparison with other treatment modalities; and afterward, the benefits disappeared. In addition, we found that the clinicopathological characteristics played central roles when assessing the effects of different treatment modalities.

The current study benefited from the large, population‐based SEER CSR 2000‐2016 database. We finally retrieved the data of 25,232 patients diagnosed with either esophageal or gastroesophageal junction cancer between January 2000 and December 2016, which minimizes information bias and provides reliable results. The detailed information on clinicopathological characteristics captures the time‐dependent effects of various treatment modalities. We focused on COS and time‐dependent effect on survival probability for patients receiving different treatment modalities. The 5‐year COS increased quickly from diagnosis within the first 3 years and slowed down afterward, as the patients with poor prognosis had died. Meanwhile, the time‐dependent HR showed that the HR reached the peak was within 2 years after diagnosis and then fell, indicating that a fraction of patients were cured or had long‐term survival after 3 years. This finding was consistent with the previous studies on patients with esophageal cancer that most recurrences occurred within 2 years after treatment, whether surgery only or preoperative chemoradiotherapy.^31^, ^32^, ^33^, ^34^ The trend of improved COS and time‐varying HR indicated the survival benefits under multimodality treatment for esophageal cancer.

Previous researches on SEER database analysis usually reported survival outcomes based on Cox proportional‐hazards models.^35^, ^36^ When the PHA is violated, the estimated HR is clinically meaningless. In contrast, our study provided a dynamic view of survival probability as time accrued and the time‐dependent effect according to the different stages. For patients with localized esophageal cancer, radical esophagectomy was the commonly used treatment approach. Although preoperative therapy was also recommended for high‐risk patients, such as those with long tumor length or poor differentiation, the application of multimodality therapy for early stage esophageal cancer was reported to result in unexpected toxicities and dissatisfactory survival outcomes.^37^, ^38^ Recent advances in surgical techniques reveal that minimally invasive thoracolaparoscopic esophagectomy is less likely to result in postoperative complications, with better functional recovery and higher quality of life compared with the traditional open transthoracic esophagectomy.^39^ The high adjusted COS in our study verified the fundamental role of surgery in localized disease. The landmark analysis yielded consistent results. The possible explanation was the relatively low recurrence rate of localized disease. Such results suggest that neoadjuvant or adjuvant therapy may not be necessary for localized disease.

Randomized controlled trials and meta‐analysis had demonstrated that preoperative chemoradiotherapy was the standard treatment for patients with regional esophageal cancer, especially for squamous cell carcinoma.^8^, ^40^, ^41^ Most of the patients (98.5%) in the preoperative radiation therapy plus surgery group in our study received chemotherapy. Our findings provided consistent results, and showed that patients with preoperative radiation therapy plus surgery had better and long‐lasting survival benefits in comparison with surgery only. This remarkable effect strongly indicated that adding preoperative therapy to surgery in patients with regional disease could substantially increase patients' prognosis. These results add evidence for the application of preoperative therapy in regional esophageal cancer. Moreover, surgery plus chemotherapy was an ideal option for regional esophageal adenocarcinoma, and postoperative radiation therapy plus surgery could improve tumor control and overall survival, which were consistent with several previous studies.^42^, ^43^ Finally, in patients with distant disease, preoperative radiation therapy plus surgery and surgery plus chemotherapy might bring short‐term benefits to primary tumor, such as patients with oligometastasis or non‐regional lymph nodes involved.^44^, ^45^ However, due to the poor prognosis of distant metastasis, the benefits of all these treatment modalities were similar. In such a case, comorbidities, quality of life, and social‐economic factors would be considered rather than intensive treatment. Targeted therapy, immunotherapy, or clinical trials also served as possible selections.

Kim et al. described the conditional survival for esophageal cancer using the SEER database between 1988 and 2011.^46^ In Kim's study, they used the Cox proportional‐hazards model to estimate conditional survival. However, we applied the IPTW method with adjusting clinicopathological covariates, which provides a more accurate estimate, especially when the PHA is violated. Moreover, we focused on the effect of various treatment modalities for patients with different stages. Considering that OS in patients with esophageal cancer decreased rapidly after 3 years of diagnosis, we focused on the 5‐year adjusted COS given patients had survived 3 years across localized, regional, and distant stages. The results provided information on dynamic survival probability changes and suggestions on treatment decisions.

Nevertheless, we acknowledge several limitations in this study. First, the detailed treatment information lacks in the SEER database, such as the time from diagnosis to surgery, radiation dose, and chemotherapy regimens. These are the common drawbacks of the retrospective or surveillance study, which limited the reliability of the study results, inducing information bias.^47^ Second, patients who did not receive any treatment had a high 5‐year adjusted COS given that the patients have already survived 3 years compared with other treatments. This is expected as the patients with poor prognosis died after the diagnosis, which is one of the advantages of the COS for describing the dynamic prognosis, although selection bias may arise. Third, the immortal‐time bias, which refers to the patients receiving any treatment modalities, has to survive to the date of treatment, may also bias our results away from the null.^48^, ^49^ However, the results derived from the landmark analysis indicated that the effect of immortal‐time bias seemed minimal.

In summary, with advanced treatment modalities, a steady increase is seen in prognosis for patients with esophageal or gastroesophageal junction cancer. In this study, we provide reliable and accurate contemporary estimates of dynamic prognosis in patients with esophageal or gastroesophageal junction cancer after receiving different treatment modalities, especially for the preoperative radiation therapy plus surgery and surgery only. Considering that the outlook of esophageal or gastroesophageal junction cancer after diagnosis remains poor, there still exists an increasing need for a deep understanding of the prognosis of these patients. In addition, primary care lead research is also required for patients with esophageal or gastroesophageal junction cancer to achieve a better prognosis.

## ETHICAL STATEMENT

Because the study used preexisting data with no personal identifiers, this study was exempt from review by the institutional review board.

## Supporting information

Table S1‐S2

## Data Availability

The data that support the findings of this study are openly available in Surveillance, Epidemiology, and End Results (SEER) Program (www.seer.cancer.gov) Research Data (1975–2016), National Cancer Institute, DCCPS, Surveillance Research Program, released April 2019, based on the November 2018 submission, reference number [20].
