# Supplementary material for: Conditional survival in patients with esophageal or gastroesophageal junction cancer after receiving various treatment modalities
Source: Cancer Med. 2020 Dec 12;10(2):659–74. doi: 10.1002/cam4.3651 (PMC7877350; doi:10.1002/cam4.3651)
Supplement: Supplementary file 1 — Table S1‐S2 [file CAM4-10-659-s001.docx]

**Conditional overall survival in patients with esophageal cancer after receiving various treatment modalities**

**Table S1.** Baseline clinicopathological characteristics and causes of death of the study participants according to the treatment modalities*

| Label | Overall | Treatment Modalities | | | | | | | |
| --- | --- | --- | --- | --- | --- | --- | --- | --- | --- |
|  |  | S | RT | CT | RT+CT | RT+S | S+RT | S+CT | None |
| N (%) | 25232 (100) | 3453 (13.7) | 1698 (6.7) | 3265 (12.9) | 7339 (29.1) | 3624 (14.4) | 1763 (7.0) | 712 (2.8) | 3378 (13.4) |
| Sex Female (%) | 5364 (21.3) | 749 (21.7) | 439 (25.9) | 601 (18.4) | 1564 (21.3) | 575 (15.9) | 356 (20.2) | 148 (20.8) | 932 (27.6) |
| Age at diagnosis, mean (SD) | 66.73 (11.9) | 68.17 (11.0) | 73.46 (12.1) | 63.34 (11.5) | 67.57 (11.3) | 61.65 (9.6) | 63.21 (11.0) | 62.04 (11.7) | 71.61 (13.0) |
| Age at diagnosis (%) |  |  |  |  |  |  |  |  |  |
| 18-49 | 1900 (7.5) | 174 (5.0) | 52 (3.1) | 376 (11.5) | 457 (6.2) | 377 (10.4) | 191 (10.8) | 96 (13.5) | 177 (5.2) |
| 50-59 | 5070 (20.1) | 585 (16.9) | 209 (12.3) | 802 (24.6) | 1325 (18.1) | 1030 (28.4) | 466 (26.4) | 183 (25.7) | 470 (13.9) |
| 60-69 | 7600 (30.1) | 1002 (29.0) | 336 (19.8) | 1093 (33.5) | 2203 (30.0) | 1435 (39.6) | 555 (31.5) | 232 (32.6) | 744 (22.0) |
| 70-79 | 6791 (26.9) | 1151 (33.3) | 459 (27.0) | 747 (22.9) | 2219 (30.2) | 718 (19.8) | 450 (25.5) | 166 (23.3) | 881 (26.1) |
| 80+ | 3871 (15.3) | 541 (15.7) | 642 (37.8) | 247 (7.6) | 1135 (15.5) | 64 (1.8) | 101 (5.7) | 35 (4.9) | 1106 (32.7) |
| Ethnicity (%) |  |  |  |  |  |  |  |  |  |
| White | 21403 (84.8) | 3063 (88.7) | 1351 (79.6) | 2815 (86.2) | 6031 (82.2) | 3334 (92.0) | 1469 (83.3) | 603 (84.7) | 2737 (81.0) |
| Black | 2064 (8.2) | 151 (4.4) | 187 (11.0) | 247 (7.6) | 778 (10.6) | 155 (4.3) | 116 (6.6) | 41 (5.8) | 389 (11.5) |
| Other | 1765 (7.0) | 239 (6.9) | 160 (9.4) | 203 (6.2) | 530 (7.2) | 135 (3.7) | 178 (10.1) | 68 (9.6) | 252 (7.5) |
| Marital status (%) |  |  |  |  |  |  |  |  |  |
| Single (never married) | 3363 (13.3) | 349 (10.1) | 237 (14.0) | 474 (14.5) | 1077 (14.7) | 399 (11.0) | 218 (12.4) | 90 (12.6) | 519 (15.4) |
| Married (including common law) | 15039 (59.6) | 2213 (64.1) | 815 (48.0) | 2058 (63.0) | 4220 (57.5) | 2550 (70.4) | 1165 (66.1) | 497 (69.8) | 1521 (45.0) |
| Other/Unknown | 6830 (27.1) | 891 (25.8) | 646 (38.0) | 733 (22.5) | 2042 (27.8) | 675 (18.6) | 380 (21.6) | 125 (17.6) | 1338 (39.6) |
| Tumor grade (%) |  |  |  |  |  |  |  |  |  |
| Well differentiated | 1138 (4.5) | 388 (11.2) | 63 (3.7) | 84 (2.6) | 246 (3.4) | 145 (4.0) | 63 (3.6) | 32 (4.5) | 117 (3.5) |
| Moderately differentiated | 8132 (32.2) | 1214 (35.2) | 534 (31.4) | 870 (26.6) | 2501 (34.1) | 1249 (34.5) | 563 (31.9) | 209 (29.4) | 992 (29.4) |
| Poorly differentiated | 11366 (45.0) | 1185 (34.3) | 741 (43.6) | 1697 (52.0) | 3221 (43.9) | 1753 (48.4) | 932 (52.9) | 426 (59.8) | 1411 (41.8) |
| Cell type not determined | 4596 (18.2) | 666 (19.3) | 360 (21.2) | 614 (18.8) | 1371 (18.7) | 477 (13.2) | 205 (11.6) | 45 (6.3) | 858 (25.4) |
| Tumor stage (%) |  |  |  |  |  |  |  |  |  |
| Localized | 6761 (26.8) | 2407 (69.7) | 442 (26.0) | 226 (6.9) | 1577 (21.5) | 709 (19.6) | 222 (12.6) | 112 (15.7) | 1066 (31.6) |
| Regional | 8777 (34.8) | 878 (25.4) | 441 (26.0) | 294 (9.0) | 2792 (38.0) | 2366 (65.3) | 985 (55.9) | 429 (60.3) | 592 (17.5) |
| Distant | 9694 (38.4) | 168 (4.9) | 815 (48.0) | 2745 (84.1) | 2970 (40.5) | 549 (15.1) | 556 (31.5) | 171 (24.0) | 1720 (50.9) |
| Histology type squamous cell cancer (%) | 6033 (23.9) | 454 (13.1) | 626 (36.9) | 518 (15.9) | 2617 (35.7) | 539 (14.9) | 321 (18.2) | 55 (7.7) | 903 (26.7) |
| Primary site of gastroesophageal junction (%) | 8429 (33.4) | 1496 (43.3) | 383 (22.6) | 1327 (40.6) | 1651 (22.5) | 1213 (33.5) | 800 (45.4) | 442 (62.1) | 1117 (33.1) |
| Year of diagnosis (%) |  |  |  |  |  |  |  |  |  |
| 2000-2004 | 7010 (27.8) | 1196 (34.6) | 532 (31.3) | 670 (20.5) | 2010 (27.4) | 797 (22.0) | 649 (36.8) | 186 (26.1) | 970 (28.7) |
| 2005-2009 | 7804 (30.9) | 1094 (31.7) | 509 (30.0) | 981 (30.0) | 2269 (30.9) | 1076 (29.7) | 545 (30.9) | 235 (33.0) | 1095 (32.4) |
| 2010-2014 | 8588 (34.0) | 966 (28.0) | 531 (31.3) | 1335 (40.9) | 2491 (33.9) | 1457 (40.2) | 467 (26.5) | 252 (35.4) | 1089 (32.2) |
| 2015-2016 | 1830 (7.3) | 197 (5.7) | 126 (7.4) | 279 (8.5) | 569 (7.8) | 294 (8.1) | 102 (5.8) | 39 (5.5) | 224 (6.6) |
| Causes of death (%)† |  |  |  |  |  |  |  |  |  |
| Alive | 4572 (18.1) | 1444 (41.8) | 52 (3.1) | 197 (6.0) | 796 (10.8) | 1336 (36.9) | 343 (19.5) | 212 (29.8) | 192 (5.7) |
| Esophagus C | 13814 (54.7) | 909 (26.3) | 1234 (72.7) | 1946 (59.6) | 4850 (66.1) | 1676 (46.2) | 879 (49.9) | 256 (36.0) | 2064 (61.1) |
| Stomach C | 2771 (11.0) | 250 (7.2) | 130 (7.7) | 766 (23.5) | 521 (7.1) | 155 (4.3) | 259 (14.7) | 161 (22.6) | 529 (15.7) |
| AAD/OAD | 38 (0.2) | 7 (0.2) | 3 (0.2) | 2 (0.1) | 14 (0.2) | 6 (0.2) | 2 (0.1) | 0 (0.0) | 4 (0.1) |
| Accidents/AEs | 89 (0.4) | 24 (0.7) | 4 (0.2) | 8 (0.2) | 22 (0.3) | 13 (0.4) | 4 (0.2) | 3 (0.4) | 11 (0.3) |
| AD | 50 (0.2) | 24 (0.7) | 3 (0.2) | 1 (0.0) | 4 (0.1) | 3 (0.1) | 4 (0.2) | 0 (0.0) | 11 (0.3) |
| CLD/Cirrhosis | 43 (0.2) | 9 (0.3) | 3 (0.2) | 3 (0.1) | 12 (0.2) | 2 (0.1) | 8 (0.5) | 1 (0.1) | 5 (0.1) |
| COPD | 235 (0.9) | 61 (1.8) | 15 (0.9) | 6 (0.2) | 83 (1.1) | 38 (1.0) | 11 (0.6) | 1 (0.1) | 20 (0.6) |
| CVA | 131 (0.5) | 42 (1.2) | 9 (0.5) | 10 (0.3) | 28 (0.4) | 8 (0.2) | 6 (0.3) | 5 (0.7) | 23 (0.7) |
| DC/Non-COD | 148 (0.6) | 12 (0.3) | 7 (0.4) | 24 (0.7) | 34 (0.5) | 16 (0.4) | 10 (0.6) | 6 (0.8) | 39 (1.2) |
| DM | 60 (0.2) | 18 (0.5) | 8 (0.5) | 3 (0.1) | 16 (0.2) | 4 (0.1) | 2 (0.1) | 0 (0.0) | 9 (0.3) |
| Female genital C | 36 (0.1) | 6 (0.2) | 5 (0.3) | 2 (0.1) | 9 (0.1) | 1 (0.0) | 3 (0.2) | 0 (0.0) | 10 (0.3) |
| HD | 861 (3.4) | 199 (5.8) | 58 (3.4) | 59 (1.8) | 273 (3.7) | 89 (2.5) | 65 (3.7) | 13 (1.8) | 105 (3.1) |
| HIV/OIPD | 55 (0.2) | 12 (0.3) | 2 (0.1) | 6 (0.2) | 22 (0.3) | 3 (0.1) | 3 (0.2) | 0 (0.0) | 7 (0.2) |
| Leukemia/Lymphoma | 48 (0.2) | 9 (0.3) | 4 (0.2) | 4 (0.1) | 9 (0.1) | 8 (0.2) | 3 (0.2) | 2 (0.3) | 9 (0.3) |
| Lung C | 277 (1.1) | 49 (1.4) | 17 (1.0) | 31 (0.9) | 77 (1.0) | 29 (0.8) | 22 (1.2) | 7 (1.0) | 45 (1.3) |
| Male genital C | 52 (0.2) | 9 (0.3) | 9 (0.5) | 1 (0.0) | 11 (0.1) | 6 (0.2) | 3 (0.2) | 3 (0.4) | 10 (0.3) |
| MMC | 430 (1.7) | 57 (1.7) | 27 (1.6) | 77 (2.4) | 124 (1.7) | 35 (1.0) | 25 (1.4) | 9 (1.3) | 76 (2.2) |
| Nephropathy | 58 (0.2) | 18 (0.5) | 5 (0.3) | 2 (0.1) | 15 (0.2) | 6 (0.2) | 5 (0.3) | 0 (0.0) | 7 (0.2) |
| Oral/Pharynx C | 82 (0.3) | 8 (0.2) | 15 (0.9) | 4 (0.1) | 31 (0.4) | 4 (0.1) | 5 (0.3) | 0 (0.0) | 15 (0.4) |
| Oth C1 | 33 (0.1) | 5 (0.1) | 5 (0.3) | 2 (0.1) | 6 (0.1) | 4 (0.1) | 6 (0.3) | 1 (0.1) | 4 (0.1) |
| Oth C2 | 18 (0.1) | 3 (0.1) | 0 (0.0) | 4 (0.1) | 7 (0.1) | 2 (0.1) | 1 (0.1) | 0 (0.0) | 1 (0.0) |
| Oth Dig C | 254 (1.0) | 35 (1.0) | 20 (1.2) | 37 (1.1) | 68 (0.9) | 12 (0.3) | 13 (0.7) | 7 (1.0) | 62 (1.8) |
| Oth Dis | 743 (2.9) | 167 (4.8) | 39 (2.3) | 45 (1.4) | 213 (2.9) | 122 (3.4) | 50 (2.8) | 21 (2.9) | 86 (2.5) |
| Oth Respir C | 50 (0.2) | 14 (0.4) | 5 (0.3) | 9 (0.3) | 11 (0.1) | 2 (0.1) | 1 (0.1) | 0 (0.0) | 8 (0.2) |
| Pneumonia/Influenza | 101 (0.4) | 24 (0.7) | 7 (0.4) | 1 (0.0) | 34 (0.5) | 14 (0.4) | 10 (0.6) | 2 (0.3) | 9 (0.3) |
| Septicemia | 90 (0.4) | 20 (0.6) | 4 (0.2) | 6 (0.2) | 26 (0.4) | 18 (0.5) | 8 (0.5) | 1 (0.1) | 7 (0.2) |
| Suicide/Injury | 55 (0.2) | 7 (0.2) | 6 (0.4) | 6 (0.2) | 15 (0.2) | 5 (0.1) | 9 (0.5) | 1 (0.1) | 6 (0.2) |
| Urinary C | 38 (0.2) | 11 (0.3) | 2 (0.1) | 3 (0.1) | 8 (0.1) | 7 (0.2) | 3 (0.2) | 0 (0.0) | 4 (0.1) |

* S: Surgery only; RT: Radiation therapy only; CT: Chemotherapy only; RT+CT: Chemoradiotherapy; RT+S: Preoperative radiation therapy plus surgery; S+RT: Surgery plus postoperative radiation therapy; S+CT: Surgery plus chemotherapy; None: no treatment.

† Esophagua C: Esophageal cancer; HD: diseases of heart; Stomach C: stomach cancer; Oth Dis: other diseases (non-cancer) of death, including unknown behavior neoplasm, tuberculosis, syphilis, hypertension without heart disease, stomach and duodenal ulcers, complications of pregnancy, childbirth, puerperium, congenital anomalies, certain conditions originating in perinatal period, symptoms, signs and ill-defined conditions, homicide and legal intervention; MMC: miscellaneous malignant cancer, including mesothelioma and kaposi sarcoma; Lung C: lung cancer; COPD: chronic obstructive pulmonary disease; Oth Dig C: other digestive system cancer, including small intestine, colon, rectum, anus, anal canal, anorectum and others; DC/non-COD: state DC not available or state DC available but no cause of death; CVA: cerebrovascular diseases; Accidents/AEs: accidents and adverse effects; HIV/OIPD: human immunodeficiency virus (HIV) and other infectious and parasitic diseases; Female genital C: female genital cancer; Oral/Pharynx C: oral/pharynx cancer; AAD/OAD: atherosclerosis, aortic aneurysm and dissection, and other diseases of arteries, arterioles, capillaries; Male genital C: male genital cancer; Oth C1: bones and joints, soft tissue (including heart), skin system cancer and myeloma; DM: diabetes mellitus; Other Respir C: other respiratory system cancer, including larynx and others; CLD/Cirrhosis: chronic liver disease and cirrhosis; AD: Alzheimer’s disease; Oth C2: eye, orbit, endocrine brain and other nervous system cancer; and Urinary C: urinary cancer.

**Table S2**. Conditional probabilities of overall survival at various time points stratified by treatment modalities after adjustment for age at diagnosis, sex, ethnicity, marital status, tumor grade and stage, histological type, tumor location, and year at diagnosis

| Time point (months) by treatment modalities* | Observed survival (%) | Conditional probability of survival (%) by time point (months) | | | | | |
| --- | --- | --- | --- | --- | --- | --- | --- |
|  |  | 36 | 48 | 60 | 72 | 84 | 96 |
| S |  |  |  |  |  |  |  |
| 12 | 0.54 (0.50-0.58) | 0.55 (0.54-0.56) | 0.48 (0.47-0.49) | 0.43 (0.42-0.44) | 0.39 (0.38-0.40) | 0.35 (0.34-0.36) | 0.32 (0.31-0.33) |
| 24 | 0.36 (0.33-0.40) | 0.82 (0.81-0.83) | 0.72 (0.71-0.73) | 0.64 (0.63-0.65) | 0.58 (0.57-0.59) | 0.51 (0.50-0.53) | 0.47 (0.46-0.49) |
| 36 | 0.30 (0.27-0.33) | 1.00 (1.00-1.00) | 0.88 (0.87-0.88) | 0.78 (0.77-0.79) | 0.71 (0.69-0.72) | 0.63 (0.61-0.64) | 57.9 (56.5-59.2) |
| 48 | 0.26 (0.24-0.29) |  | 1.00 (1.00-1.00) | 0.89 (0.88-0.90) | 0.80 (0.79-0.82) | 0.72 (0.70-0.73) | 0.66 (0.65-0.67) |
| 60 | 0.23 (0.21-0.26) |  |  | 1.00 (1.00-1.00) | 0.90 (0.90-0.91) | 0.81 (0.79-0.82) | 0.74 (0.73-0.76) |
| RT |  |  |  |  |  |  |  |
| 12 | 0.23 (0.20-0.26) | 0.33 (0.32-0.34) | 0.22 (0.21-0.23) | 0.18 (0.17-0.19) | 0.14 (0.13-0.15) | 0.12 (0.11-0.13) | 0.11 (0.10-0.12) |
| 24 | 0.11 (0.09-0.13) | 0.69 (0.67-0.71) | 0.45 (0.43-0.47) | 0.37 (0.35-0.39) | 0.30 (0.28-0.32) | 0.26 (0.24-0.28) | 0.23 (0.21-0.25) |
| 36 | 0.08 (0.06-0.10) | 1.00 (1.00-1.00) | 0.66 (0.63-0.68) | 0.54 (0.51-0.56) | 0.44 (0.41-0.46) | 0.37 (0.35-0.40) | 33.8 (31.3-36.4) |
| 48 | 0.05 (0.04-0.07) |  | 1.00 (1.00-1.00) | 0.82 (0.79-0.85) | 0.66 (0.63-0.70) | 0.57 (0.53-0.60) | 0.51 (0.48-0.55) |
| 60 | 0.04 (0.03-0.06) |  |  | 1.00 (1.00-1.00) | 0.81 (0.78-0.84) | 0.69 (0.65-0.72) | 0.63 (0.59-0.66) |
| CT |  |  |  |  |  |  |  |
| 12 | 0.35 (0.32-0.38) | 0.23 (0.22-0.24) | 0.19 (0.18-0.20) | 0.14 (0.14-0.15) | 0.12 (0.11-0.12) | 0.09 (0.08-0.09) | 0.07 (0.06-0.08) |
| 24 | 0.16 (0.14-0.19) | 0.50 (0.49-0.52) | 0.42 (0.40-0.44) | 0.31 (0.30-0.33) | 0.26 (0.24-0.27) | 0.19 (0.17-0.20) | 0.15 (0.14-0.17) |
| 36 | 0.08 (0.06-0.10) | 1.00 (1.00-1.00) | 0.83 (0.81-0.85) | 0.62 (0.60-0.65) | 0.51 (0.48-0.53) | 0.37 (0.34-0.40) | 29.9 (27.3-32.6) |
| 48 | 0.07 (0.05-0.09) |  | 1.00 (1.00-1.00) | 0.75 (0.72-0.77) | 0.61 (0.58-0.64) | 0.45 (0.41-0.48) | 0.36 (0.33-0.39) |
| 60 | 0.05 (0.03-0.07) |  |  | 1.00 (1.00-1.00) | 0.82 (0.79-0.84) | 0.60 (0.56-0.63) | 0.48 (0.44-0.52) |
| RT+CT |  |  |  |  |  |  |  |
| 12 | 0.47 (0.46-0.48) | 0.36 (0.35-0.37) | 0.28 (0.27-0.29) | 0.22 (0.21-0.23) | 0.18 (0.17-0.19) | 0.15 (0.15-0.16) | 0.14 (0.13-0.15) |
| 24 | 0.25 (0.24-0.27) | 0.67 (0.66-0.69) | 0.52 (0.50-0.53) | 0.41 (0.40-0.43) | 0.34 (0.32-0.35) | 0.29 (0.27-0.30) | 0.26 (0.24-0.27) |
| 36 | 0.17 (0.16-0.18) | 1.00 (1.00-1.00) | 0.77 (0.75-0.78) | 0.61 (0.60-0.63) | 0.50 (0.48-0.52) | 0.43 (0.41-0.44) | 38.2 (36.3-40.0) |
| 48 | 0.13 (0.12-0.14) |  | 1.00 (1.00-1.00) | 0.80 (0.78-0.82) | 0.65 (0.63-0.67) | 0.56 (0.53-0.58) | 0.50 (0.47-0.52) |
| 60 | 0.11 (0.10-0.11) |  |  | 1.00 (1.00-1.00) | 0.82 (0.80-0.83) | 0.69 (0.67-0.72) | 0.62 (0.60-0.65) |
| RT+S |  |  |  |  |  |  |  |
| 12 | 0.75 (0.72-0.79) | 0.61 (0.60-0.61) | 0.52 (0.51-0.53) | 0.47 (0.46-0.48) | 0.44 (0.43-0.45) | 0.38 (0.38-0.39) | 0.36 (0.35-0.37) |
| 24 | 0.55 (0.51-0.59) | 0.83 (0.83-0.84) | 0.71 (0.70-0.72) | 0.64 (0.64-0.65) | 0.60 (0.59-0.61) | 0.53 (0.52-0.54) | 0.50 (0.49-0.51) |
| 36 | 0.46 (0.42-0.50) | 1.00 (1.00-1.00) | 0.85 (0.85-0.86) | 0.77 (0.77-0.78) | 0.72 (0.71-0.73) | 0.63 (0.62-0.64) | 59.9 (58.7-61.0) |
| 48 | 0.39 (0.35-0.43) |  | 1.00 (1.00-1.00) | 0.91 (0.90-0.91) | 0.85 (0.84-0.85) | 0.74 (0.73-0.75) | 0.70 (0.69-0.71) |
| 60 | 0.35 (0.31-0.40) |  |  | 1.00 (1.00-1.00) | 0.93 (0.93-0.94) | 0.82 (0.81-0.83) | 0.77 (0.76-0.78) |
| S+RT |  |  |  |  |  |  |  |
| 12 | 0.65 (0.62-0.68) | 0.52 (0.51-0.52) | 0.42 (0.41-0.43) | 0.35 (0.34-0.36) | 0.30 (0.29-0.31) | 0.27 (0.26-0.28) | 0.25 (0.24-0.26) |
| 24 | 0.42 (0.39-0.46) | 0.79 (0.78-0.80) | 0.65 (0.64-0.66) | 0.54 (0.53-0.55) | 0.46 (0.45-0.47) | 0.41 (0.40-0.42) | 0.38 (0.37-0.39) |
| 36 | 0.33 (0.30-0.37) | 1.00 (1.00-1.00) | 0.82 (0.81-0.83) | 0.68 (0.67-0.69) | 0.58 (0.57-0.59) | 0.52 (0.51-0.54) | 48.0 (46.7-49.3) |
| 48 | 0.27 (0.25-0.31) |  | 1.00 (1.00-1.00) | 0.83 (0.82-0.84) | 0.71 (0.70-0.72) | 0.64 (0.62-0.65) | 0.59 (0.57-0.60) |
| 60 | 0.23 (0.20-0.26) |  |  | 1.00 (1.00-1.00) | 0.86 (0.84-0.87) | 0.77 (0.75-0.78) | 0.71 (0.69-0.72) |
| S+CT |  |  |  |  |  |  |  |
| 12 | 0.69 (0.63-0.75) | 0.56 (0.56-0.57) | 0.43 (0.42-0.44) | 0.36 (0.36-0.37) | 0.34 (0.33-0.35) | 0.27 (0.27-0.28) | 0.22 (0.22-0.23) |
| 24 | 0.52 (0.45-0.58) | 0.75 (0.74-0.76) | 0.57 (0.56-0.58) | 0.48 (0.47-0.49) | 0.45 (0.44-0.46) | 0.36 (0.35-0.37) | 0.30 (0.29-0.31) |
| 36 | 0.39 (0.33-0.46) | 1.00 (1.00-1.00) | 0.76 (0.75-0.77) | 0.64 (0.63-0.66) | 0.60 (0.59-0.61) | 0.48 (0.47-0.50) | 39.9 (38.6-41.1) |
| 48 | 0.30 (0.24-0.36) |  | 1.00 (1.00-1.00) | 0.85 (0.84-0.85) | 0.79 (0.78-0.80) | 0.63 (0.62-0.65) | 0.52 (0.51-0.54) |
| 60 | 0.25 (0.20-0.31) |  |  | 1.00 (1.00-1.00) | 0.94 (0.93-0.94) | 0.75 (0.74-0.76) | 0.62 (0.60-0.63) |
| None |  |  |  |  |  |  |  |
| 12 | 0.21 (0.19-0.23) | 0.45 (0.44-0.47) | 0.37 (0.35-0.38) | 0.34 (0.33-0.36) | 0.32 (0.30-0.33) | 0.29 (0.28-0.31) | 0.28 (0.27-0.30) |
| 24 | 0.12 (0.10-0.13) | 0.81 (0.79-0.82) | 0.65 (0.63-0.67) | 0.61 (0.59-0.63) | 0.56 (0.54-0.58) | 0.52 (0.50-0.54) | 0.50 (0.48-0.52) |
| 36 | 0.09 (0.08-0.11) | 1.00 (1.00-1.00) | 0.81 (0.79-0.83) | 0.76 (0.74-0.78) | 0.70 (0.68-0.72) | 0.64 (0.62-0.67) | 62.2 (59.7-64.6) |
| 48 | 0.08 (0.07-0.09) |  | 1.00 (1.00-1.00) | 0.94 (0.92-0.95) | 0.86 (0.84-0.88) | 0.80 (0.77-0.82) | 0.77 (0.74-0.79) |
| 60 | 0.07 (0.06-0.09) |  |  | 1.00 (1.00-1.00) | 0.92 (0.90-0.94) | 0.85 (0.83-0.87) | 0.82 (0.80-0.84) |

* S: Surgery only; RT: Radiation therapy only; CT: Chemotherapy only; RT+CT: Chemoradiotherapy; RT+S: Preoperative radiation therapy plus surgery; S+RT: Surgery plus postoperative radiation therapy; S+CT: Surgery plus chemotherapy; None: no treatment.
